# Hair analysis for the biomonitoring of pesticide exposure: comparison with blood and urine in a rat model

**DOI:** 10.1007/s00204-016-1910-9

**Published:** 2016-12-23

**Authors:** Brice M. R. Appenzeller, Emilie M. Hardy, Nathalie Grova, Caroline Chata, François Faÿs, Olivier Briand, Henri Schroeder, Radu-Corneliu Duca

**Affiliations:** 1 Human Biomonitoring Research Unit, Department of Population Health, Luxembourg Institute of Health, 29 Rue Henri Koch, 4354 Esch-sur Alzette, Luxembourg; 2 Competence Center in Methodology and Statistics, Luxembourg Institute of Health, 29 Rue Henri Koch, 4354 Esch-sur Alzette, Luxembourg; 3 French Ministry of Agriculture, Agrifood, and Forestry, Paris, France; 4 Unit Research Animal and Functionality of Animal Products (URAFPA), French National Institute for Agricultural Research (INRA) UC340, University of Lorraine, Nancy, France

**Keywords:** Hair analysis, Plasma, Urine, Pesticide, Biomonitoring, Exposure

## Abstract

Urine and plasma have been used to date for the biomonitoring of exposure to pollutants and are still the preferred fluids for this purpose; however, these fluids mainly provide information on the short term and may present a high level of variability regarding pesticide concentrations, especially for nonpersistent compounds. Hair analysis may provide information about chronic exposure that is averaged over several months; therefore, this method has been proposed as an alternative to solely relying on these fluids. Although the possibility of detecting pesticides in hair has been demonstrated over the past few years, the unknown linkage between exposure and pesticides concentration in hair has limited the recognition of this matrix as a relevant tool for assessing human exposure. Based on a rat model in which there was controlled exposure to a mixture of pesticides composed of lindane, β-hexachlorocyclohexane, β-endosulfan, p,p′-DDT, p,p′-DDE, dieldrin, pentachlorophenol, diazinon, chlorpyrifos, cyhalothrin, permethrin, cypermethrin, propiconazole, fipronil, oxadiazon, diflufenican, trifluralin, carbofuran, and propoxur, the current work demonstrates the association between exposure intensity and resulting pesticide concentration in hair. We also compared the results obtained from a hair analysis to urine and plasma collected from the same rats. Hair, blood, and urine were collected from rats submitted to 90-day exposure by gavage to the aforementioned mixture of common pesticides at different levels. We observed a linear relationship between exposure intensity and the concentration of pesticides in the rats’ hair (*R*_Pearson_ 0.453–0.978, *p* < 0.01). A comparison with results from urine and plasma samples demonstrated the relevance of hair analysis and, for many chemicals, its superiority over using fluids for differentiating animals from different groups and for re-attributing animals to their correct groups of exposure based on pesticide concentrations in the matrix. Therefore, this study strongly supports hair analysis as a reliable tool to be used during epidemiological studies to investigate exposure-associated adverse health effects.

**Electronic supplementary material:**

The online version of this article (doi:10.1007/s00204-016-1910-9) contains supplementary material, which is available to authorized users.

## Introduction

Both the adverse effects of pesticides and the ubiquity of human exposure to these chemicals have been documented by an increasing number of data sets (Ntzani et al. 2013). Although different approaches can be used to assess exposure, biomonitoring (detecting chemicals and/or their metabolites in biological matrices) often remains as the preferred approach because it offers the advantage of integrating all of the possible sources and routes of exposure and of representing the internal dose from exposure. Correctly and accurately assessing the level of exposure to organic pollutants such as pesticides, however, remains challenging. Indeed, mainly because of logistical and financial constraints, an exposure assessment most often relies on a single biological sample per individual, typically blood or urine, the latter being generally preferred because urine is collected in a noninvasive manner (Esteban and Castaño 2009). Nevertheless, for most compounds, rapid elimination from urine between successive exposure episodes results in short temporal windows of detection [defined as the timeframe within which a compound can be detected since exposure occurred (i.e., before sampling takes place and before the measured concentration is determined)] and in high variability in urinary concentration of chemicals (Attflied et al. 2014; Aylward et al. 2014). For instance, the within-subjects variability in the concentration of organophosphorus and pyrethroid urinary metabolites measured in children from the Seattle, WA, area followed over 1 year was reported to be 2–11 times higher than the between-individual variability (Attflied et al. 2014). Moreover, it was demonstrated that a single urine sample was clearly insufficient to consistently categorize children’s exposure into quartiles (Attflied et al. 2014). The variability associated with chemical concentrations in biological fluids, especially for short half-life chemicals, increases the risk of misclassification of individuals with regard to their exposure levels, and this results in a dramatic loss in statistical power in a study of associated adverse health effects.

To overcome the limitations associated with conventional biological matrices, novel approaches based on alternative matrices such as hair have been suggested. For instance, a paper (Appenzeller and Tsatsakis 2012) reviewed several publications that reported the possibility of detecting organic pollutants from different chemical classes in hair, reflecting individuals’ environmental or occupational exposure. Hair samples can be collected in a noninvasive manner and can be easily stored. However, the main advantage associated with this matrix lies in the possibility to reach extended windows of detection that may represent up to several months of exposure, depending on the length of the sample. Contrary to biological fluids such as urine and blood, the concentration of chemicals detected in hair is not influenced by short-term variations in the exposure. Instead, the concentration corresponds to an individual’s average level of exposure, which is the most relevant information for investigating possible linkages with biological effects. Although hair was reportedly used during the 1950s through 1970s to analyze for metals and was employed during the 1970s through the 1980s to determine drugs of abuse, using this matrix for detecting organic pollutants has been delayed by several limitations. For instance, there was a lack of sensitive analytical methods to sufficiently and properly monitor environmental exposure (Appenzeller 2015). This issue has, however, been resolved because considerable efforts were made during the past few years that now enable the methods to reach sensitivity levels that stand between 1000 and 10,000 times below those obtained 10 years ago (Appenzeller 2015; Salquebre et al. 2012). The influence of pigmentation on the incorporation of chemicals in hair has also been investigated, but a consistent conclusion has not yet been reached on this topic. For instance, although some studies suggested that melanin influences the concentration of some illicit and medical drugs in human and animal hair (Appenzeller and Tsatsakis 2012), no or limited influence of pigmentation was observed for ethylglucuronide, phase II metabolite of ethanol (Appenzeller et al. 2007a; Kharbouche et al. 2010), and for metabolites of polycyclic aromatic hydrocarbons (Grova et al. 2013). The main criticism of using hair analysis for the biomonitoring of pollutant exposures involves the representativeness of the level of exposure. Because qualitative results (presence or absence of drugs) are often sufficient in medico-legal contexts to prove consumption, the linkage between intake intensity and resulting concentration in hair has been poorly investigated in the past. The proportional relationship has, however, been demonstrated between ethanol consumption and the concentration of its metabolite ethyl glucuronide in hair for both humans (Appenzeller et al. 2007a) and rats (Kharbouche et al. 2010). Regarding organic pollutants, promising results have already demonstrated that rabbits exposed to a high dose of pesticides presented higher concentrations of chemicals in hair than those exposed to a low dose for organophosphates (Maravgakis et al. 2012; Margariti and Tsatsakis 2009; Tutudaki et al. 2003) and cypermethrin (pyrethroid) (Kavvalakis et al. 2014). Nevertheless, studies investigating the linkage between exposure and pesticide concentration in hair that cover a wide range of concentration levels of several chemicals are still needed so hair analysis will be recognized as a reliable marker for assessing the intensity of exposures.

In the current study, we investigated the linkage between the exposure level and the resulting concentration of pesticides and their metabolites in hair. We used rats during the study and controlled the exposure to a mixture of 19 pesticides from different chemical classes at eight different doses over a 90-day period. We compared the association between exposure level and the concentration of chemicals in hair collected at the end of the experiment to plasma and urinary concentrations collected from the same animals. For the three matrices, we also assessed the difference in pesticide concentrations between the different groups of exposure and the possibility to back determine the animals’ level of exposure on the basis of pesticide concentrations in hair, urine, and plasma.

## Materials and methods

### Animal experimentation

#### Animal housing

Sixty-eight bicolor (white and black hair) female Long-Evans rats (180–200 g, Elevage Janvier, St. Berthevin, France) were housed two per cage in a regulated environment (temperature 22 ± 3 °C; relative humidity 55 ± 10%) under a reversed light–dark cycle (lights on from 7:00 p.m. to 7:00 a.m.). Food (Teklad Global Diet 2016, Harlan, Gannat, France) and water were available ad libitum. The rats were acclimatized to the animal facility for 2 weeks before the experiment began. To minimize the external contamination of hair by pesticides from urine excretion, special bedding [with a high water-binding capacity (372%), Lignocel ¾, Harlan, Gannat, France] was replaced twice per week. Moreover, to evaluate the potential external contamination because of urine excretion, the bedding of the highest level exposed group was placed into a sentinel cage that containing four non-treated rats, which were analyzed at the end of the experiment. Feces were removed prior to placing the sentinel rats on the soiled bedding, which was replaced twice per week for the entire duration of the experiment. The analysis of the sentinel rats’ hair did not demonstrate contamination of hair because of the bedding material. The procedures applied were in compliance with the rules provided by the European Union (2010/63/EU) and were approved and supervised by the Institutional Ethics Committee of the University of Lorraine (authorization number B 54-547-13).

#### Animal treatment

Eight rats were randomly assigned to each of the experimental groups. A low-calorie water gel that contained the pesticides was administered via gavage to these rats three times per week for 90 days. The doses of the pesticides mixture used for exposure were 4, 10, 20, 40, 100, 200, and 400 µg/kg of body weight. The exposure range was set as follows: The lowest dose was the lowest level allowing the detection of pesticides in hair after a 90-day exposure, based on preliminary experiments not detailed here. Testing lower levels would not have been relevant because some compounds would not be detected anymore (analytical limitation). In addition, for some other compounds, no differences were detected in the current experiment between the lowest level of exposure and the background exposure of the controls. The highest dose was set according to toxicity of compounds, which corresponded to 1/20 of the lowest LD50 (carbofuran). The animals were weighed before each administration in order to adapt the amount of pesticide-containing mixture to the animals’ weight.

#### Pesticide gavage mixture

Pesticide mixture stock solution was prepared in ethanol every 2 weeks. Gels were prepared by mixing hot HydraGel and MediGel Sucralose (1/1, v/v, Bio Services, Uden, Netherlands), pouring the mixture into aluminum molds, and then allowing it to cool at room temperature. The gels were supplemented with the appropriate volume of pesticide mixture stock solution. Ethanol was allowed to dry at room temperature (~25 °C) until complete evaporation (i.e., minimum of 4 h). A second layer of gel was then deposited to trap the pesticides inside the gel. The control rats were fed with the same gel that was free of the pesticides. The optimal evaporation time was previously determined on gels supplemented with ethanol. The ethanol content was then assessed at different time points by using a headspace sampler coupled gas chromatography–mass spectrometry (GC–MS) instrument (Agilent Technologies, Diegem, Belgium).

#### Samples collection

The animals were shaved before the experiment began to ensure that the hair collected at the end of the study accurately reflected the 90-day period of exposure. White and black hairs were collected separately, placed in aluminum foil, and stored at −20 °C until analysis. Blood was collected in EDTA tube from the tail vein, 3 h after oral administration on Day 90. Each sampling (500–750 µL) was immediately centrifuged at 5000×*g* for 3 min at room temperature, and plasma was separated and stored at −80 °C before analysis. For urine collection, the rats were placed in individual metabolic cages (Type 304 stainless steel, Techniplast, Zwaag, Netherlands) immediately after gavage for 24 h (from Day 88 to Day 89). The urine was collected in refrigerated tubes over a 24-h period and was weighed before storage at −20 °C.

### Pesticides and analysis

The list of pesticides to which the animals were exposed included compounds from different chemical classes (see Table S1), covering a wide range of different physicochemical properties to confirm that the association between exposure and the resulting concentration in hair was not limited to a specific category of chemicals (Chata et al. 2016). The list of pesticides included chemicals classically investigated in humans (e.g., organochlorines, organophosphates, pyrethroids) in order to allow for a comparison with data obtained from the literature for humans, as well as pesticides that have been investigated less or not at all in humans.

Depending on the compounds, only parent pesticides, only metabolites, or both parent and metabolites were analyzed in the biological matrices. Details about the target chemicals are provided in Table S1. The chemicals were analyzed as previously described (Chata et al. 2016; Hardy et al. 2015). Despite the different nature of the biological matrices analyzed (liquid vs. solid), method protocols were developed to ensure similarity so they could be reliably compared to the information obtained from each matrix, thereby ensuring that differences were not likely attributable to analytical bias. Moreover, the sensitivity of the methods used proved to be quite satisfactory with regard to the literature (comparable to the best performances in the field); this ensured that the differences between matrices were not because of a lack of sensitivity (Hardy et al. 2015). Because analytical background noise was absent from the chromatograms because tandem mass spectrometry was used, the approaches based on background noise for determining the limit of detection (LOD) were not applicable. The LOD was therefore determined as the lowest concentration that was detected in the samples analyzed during this study. Selectivity was ensured by analyte retention time and by the quantification transition to confirmation transition ratio that had to be lower than the 20% difference from the ratio obtained with standard compounds. The LOD ranged from 0.02 pg/mg for β-endosulfan to 2.7 pg/mg for dichlorodiphenyltrichloroethane (p,p′-DDT) in hair, from 0.2 pg/mL for trifluralin to 13.6 ng/mL for 2-isopropoxyphenol (2-IPP) in urine, and from 2.4 pg/mL for β-endosulfan to 679 pg/mL for p,p′-DDT in plasma. Because the analysis of parent pesticides and metabolites was conducted on the same hair sample, the addition of parent organophosphate isotope-labeled analog standards was not possible because their degradation into non-labeled dialkyl phosphate (DAP) during the analytical procedure would have hindered the analysis of DAP due to exposure. In hair, only organophosphate metabolites (not parents) were therefore quantified, and only qualitative results were obtained for parents.

### Statistical analysis

The association between the level of exposure and the analyte concentration in the matrix (i.e., hair, urine, and plasma) was assessed by the Pearson product-moment correlation coefficient (*R*_Pearson_). The global tendency to present different analyte concentrations in the matrix for different levels of exposure was assessed by the Spearman’s correlation coefficient on ranks (*R*_Spearman_). The values calculated for slope, *R*_Pearson_, and *R*_Spearman_ only took into account samples with detected concentrations and included control animals when the target compounds were detected in the samples collected from them (e.g., lindane).

Inter-group differences in analyte concentration in the matrices were furthermore tested with a *t* test or a Mann–Whitney Rank Sum test when normality (Shapiro–Wilk) or equal variance test failed (SigmaPlot 12.0). For the inter-group difference statistical testings, the value of ½ LOD was attributed to the samples with a non-detected concentration. The differences between groups presented in Figs. S2 through S4 were tested as follows: top–down, the highest exposure group (400 µg/kg) was compared to the group just below (200 µg/kg). If a significant difference was observed, then it was marked with brackets, and the difference was then tested between the 200 and the 100 µg/kg groups. If no significant difference was observed between the 400 and the 200 µg/kg groups, then the 400 µg/kg group was first compared with the 100 µg/kg group, and then with groups of lower exposure if no difference was observed. The procedure was then repeated all along the groups and was then re-applied down–top (starting with the control group) in order to test all the groups.

To investigate to what extent the results obtained from hair, urine, and plasma analyses may help to accurately categorize individuals according to their level of exposure, a reverse classification analysis (RCA) was conducted based on the approach described for humans (Attflied et al. 2014). For a set of five randomly selected animals, three ascending sorts were conducted based on pesticide concentrations in hair, urine, and plasma, respectively, and then were compared with a classification according to the animals’ level of exposure used as a reference. For each matrix, one point was added if matrix-based classification was correct (cases of equality in doses were also considered), and no point was added if it was different. The procedure was reiterated 10,000 times, and the percentage of correct classification for each matrix and each compound is presented in Fig. 2. RCA was not conducted for chemicals detected in less than four groups.

## Results

For all of the chemicals (parent and metabolites) that were detected in hair or urine, the concentration in the matrix was significantly associated with the level of exposure (*p* < 0.01). In plasma, only diethyl phosphate (DEP) and diethylthiophosphate (DETP) were not significantly associated with the level of exposure, although these metabolites were always present. Slopes (linear fit) of analyte concentration in hair (pg/mg) versus level of exposure varied from 1.11 for trifluralin to 612 for β-hexachlorocyclohexane (β-HCH) (Table 1). Considerable differences in the slope “concentration in the matrix” versus the “level of exposure” between the different pesticides were also observed for urine and plasma.

**Table 1:** Association between the level of exposure and the concentration of chemicals in hair, urine, and plasma

Compounds	Hair				Urine				Plasma			
	Slope^a^	*R*_Pearson_	*R*_Spearman_ on ranks	Concentration range (pg/mg)	Slope^a^	*R*_Pearson_	*R*_Spearman_ on ranks	Concentration range (ng/mL)	Slope^a^	*R*_Pearson_	*R*_Spearman_ on ranks	Concentration range (ng/mL)
Organochlorines												
γ-HCH	119.8	0.974	0.988	1.73^b^–48.1	5 715	0.538	0.881	0.12^b^–2.02	47.2	0.895	0.968	0.93^b^–18.8
β-HCH	612.6	0.950	0.988	4.16^b^–238	6 297	0.930	0.978	0.37^b^–2.38	199	0.944	0.985	1.55^b^–77.6
β-Endosulfan	2.71	0.887	0.919	0.11^b^–1.22	–	–	–	ND	1.50	0.735	0.733	0.10^b^–0.66
p,p′-DDT	184.7	0.963	0.970	3.69^c^–74.3	NA	NA	NA	0.064^e^–0.086	175	0.891	0.968	1.32^b^–70.2
p,p′-DDE	273.5	0.956	0.987	2.73^b^–110	NA	NA	NA	0.067^e^–0.116	289	0.929	0.965	2.42^b^–112
p,p′-DDD	24.0	0.952	0.903	1.66^d^–10.2	–	–	–	ND	13.1	0.757	0.832	0.602^d^–5.87
Dieldrin	427.8	0.978	0.981	3.32^b^–160	2 069	0.654	0.900	0.023^b^–0.844	305	0.919	0.966	4.37^b^–120
Pentachlorophenol	169.0	0.955	0.988	2.24^b^–68.2	82 841	0.774	0.953	1.09^b^–32.4	2039	0.942	0.962	21.1^b^–817
Organophosphates												
Diazinon	–	–	–	ND	–	–	–	ND	–	–	–	ND
Chlorpyrifos	–	–	–	NA	–	–	–	ND	1.24	0.540	0.852	0.16^b^–0.41
DEP	4.54	0.814	0.896	0.43^b^–2.16	2.0 E + 06	0.819	0.961	18.6^b^–699	6.25	−0.208	−0.344	2.29^f^–7.89^c^
DETP	10.47	0.904	0.965	0.19^b^–4.07	9.9 E + 05	0.827	0.971	24.6^b^–623	3.26	0.172	0.528	0.16^d^–3.74^b^
TCPy	85.6	0.799	0.587	2.77^b^–34.6	2.5 E + 06	0.825	0.969	34.0^b^–914	109	0.754	0.851	5.69^b^–45.2
Pyrethroids												
Permethrin	–	–	–	ND	–	–	–	ND	1.04	0.523	0.447	0.23^g^–0.51
λ-Cyhalothrin	5.050	0.857	0.897	0.16^c^–1.73	–	–	–	ND	116	0.831	0.920	0.25^b^–43.4
Cypermethrin	NA	NA	NA	0.67^e^–1.25	–	–	–	ND	10.1	0.814	0.913	0.10^c^–3.89
Cl_2_CA	49.20	0.852	0.942	0.67^b^–19.8	1.0 E + 06	0.764	0.887	35.9^b^–443	139	0.874	0.942	1.47^b^–55.1
3-PBA	7.08	0.896	0.921	0.56^b^–3.23	1.5 E + 05	0.753	0.958	3.55^b^–56.7	422	0.850	0.932	6.54^b^–169
ClCF_3_CA	5.22	0.667	0.790	0.29^b^–2.51	1.6 E + 05	0.348	0.759	16.4^b^–73.4	9.79	0.674	0.739	0.99^b^–5.02
Carbamates												
2-IPP	26.3	0.850	0.674	0.89^b^–11.6	1.0 E + 06	0.822	0.945	19.4^b^–459	10.4	0.721	0.554	0.18^d^–4.75
Carbofuran phenol	35.10	0.931	0.469	1.14^b^–16.3	1.0 E + 06	0.817	0.940	28.8^b^–533	1.73	0.694	0.743	0.03^c^–0.71
Others												
Fipronil	31.5	0.912	0.982	0.49^b^–12.9	2 016	0.895	0.979	0.007^f^–0.81	570	0.870	0.940	0.66^b^–22.8
Fipronil sulfone	772	0.975	0.991	4.58^b^–306	10 650	0.971	0.972	0.009^b^–4.27	976	0.962	0.988	8.31^b^–388
Trifluralin	1.107	0.956	0.913	0.12^b^–0.60	4	0.506	0.429	0.0006^b^–0.002	1.57	0.830	0.910	0.09^b^–0.72
Diflufenican	35.94	0.973	0.989	0.35^b^–14.7	99	0.801	0.912	0.008^c^–0.049	15.2	0.915	0.967	0.10^b^–5.94
Oxadiazon	11.45	0.948	0.903	0.29^b^–4.85	–	–	–	ND	38.5	0.804	0.907	0.51^b^–15.0
Propiconazole	6.47	0.453	0.513	0.32^b^–3.83	–	–	–	ND	–	–	–	ND

Slope, *R*_Pearson_, and *R*_Spearman_ were calculated from detected values only (from level 40 for cypermethrin and from level 20 for λ-cyhalothrin) and were not calculated when the target compound was detected in less than four levels of exposure (indicated as “NA”)

*NA* not applicable, *ND* not detected

^a^Linear fit. For the calculation of the slope, the level of exposure was expressed as mg/kg per day, and the concentration in the matrix was expressed as pg/mg for hair and as ng/mL for urine and plasma

^b^Average concentration measured in animals exposed to 4 µg/kg

^c^Average concentration measured in animals exposed to 20 µg/kg

^d^Average concentration measured in animals exposed to 40 µg/kg

^e^Average concentration measured in animals exposed to 200 µg/kg

^f^Average concentration measured in animals exposed to 10 µg/kg

^g^Average concentration measured in animals exposed to 100 µg/kg

### Organochlorines

**Fig. 1:**
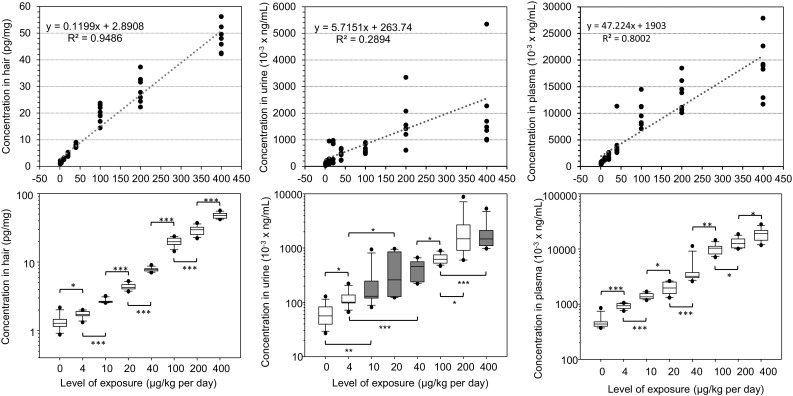
Lindane (γ-hexachlorohexane) concentrations in hair (*left*), urine (*center*), and plasma (*right*) of rats submitted to the different levels of exposure. The *top panels* present the proportional *x*-axes and provide details about each animal separately. In the urine chart, a point at 9000 pg/mL for the dosage of 200 µg/kg is not presented (out of scale) for better visibility. The *bottom panels* present *box plots*, with the *bottom* of the *box* representing the 25th quartile and the *top* of the *box* representing the 75th quartile. The *line* within the *box* represents the median, and the *whiskers* reach at a maximum of 1.5 times the interquartile range. *Circles* represent outliers. *White boxes* mean that there is a significant difference (*p* ≤ 0.05) in chemical concentrations in hair with the preceding level of exposure. *Light gray* denotes that there is a marginally significant difference (*p* value slightly greater than 0.05), and *dark gray* means that there is no significant difference with the preceding level of exposure. *p < 0.05, **p < 0.01, and ***p < 0.001

**Fig. 2:**
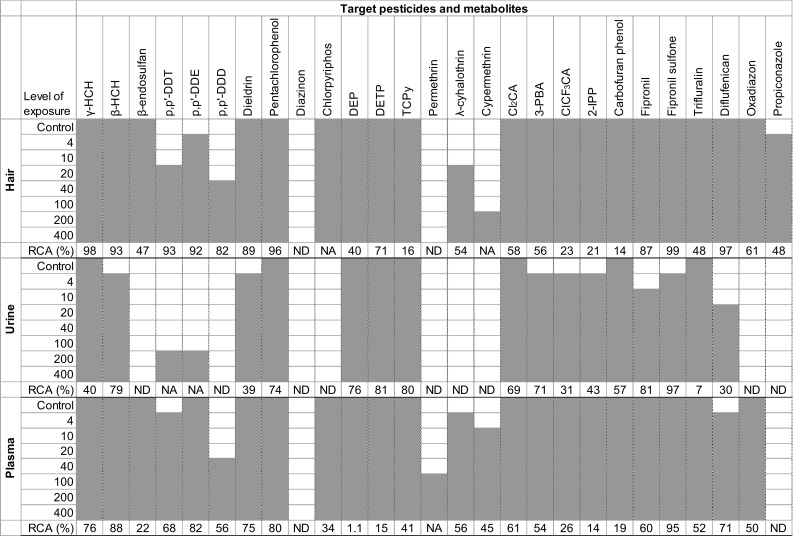
Pesticides and metabolites detected in hair, urine, and plasma according to the animals’ levels of exposure (*gray cells* denote positive detection) and reverse classification analysis (RCA) results, expressed as the percentage of correct classification based on the concentration in the matrix (*Note*: *NA* not applicable, *ND* not detected)

Organochlorines were detected in both hair and plasma at most of the levels of exposure, including the controls. In hair, exceptions were only observed for p,p′-dichlorodiphenyldichloroethylene (p,p′-DDE), which was not detected in controls, and p,p′-DDT and p,p′-dichlorodiphenyldichloroethane (p,p′-DDD), which were only detected from levels of 20 and 40 µg/kg per day, respectively. In plasma, p,p′-DDT was not detected in control animals, and its metabolite, p,p′-DDD, was detected from 40 µg/kg. In contrast, urine presented the highest rate of “undetected” (Fig. 2), and only γ-HCH (γ-hexachlorocyclohexane/lindane) and pentachlorophenol were detected in all the groups. In addition, p,p′-DDT and p,p′-DDE were only detected from the 200 µg/kg exposure level, and β-endosulfan and p,p′-DDD were not detected at all in urine. As presented with the example of lindane (γ-hexachlorohexane) (Fig. 1, box plot), significant inter-group differences in the concentration in animals’ hair were observed for all of the organochlorines, with the exception of β-endosulfan, in which a significant difference was observed between the high exposure groups only, and p,p′-DDT and p,p′-DDD, which were only detected from the exposure levels of 20 and 40 µg/kg per day, respectively (Fig. S2). In that regard, the behavior of organochlorines in plasma was very similar to what was observed in hair, although the inter-group difference was always slightly poorer in plasma. In urine, both the association between the level of exposure and the concentration in the matrix (assessed by *R*_Pearson_) and the inter-group difference (assessed by *R*_Spearman_ on ranks) were systematically lower than in hair and in plasma (Table 1). At equal levels of exposure, β-endosulfan presented the lowest concentration among all the organochlorines in both hair and plasma and was not detected in urine. The highest concentration was observed for β-HCH in hair and for pentachlorophenol in both urine and plasma.

### Organophosphates

As previously mentioned in the “Materials and methods” section of this manuscript, the methodology allowed the quantification of parent organophosphates in urine and plasma, and only provided qualitative results in hair. No parent organophosphates were detected in urine. In plasma, among the two organophosphates administered to animals, only chlorpyrifos was detected, with a mean concentration ranging from 0.15 ± 0.01 ng/mL in the control group to 0.41 ± 0.09 ng/mL in the most exposed group. Metabolites of organophosphates (DEP, DETP, and TCPy: 3,5,6-trichloro-2-pyridinol) were detected in the three matrices, whatever the level of exposure, including the controls. A significant association was always observed between the metabolite concentration in the matrix and the level of exposure, with the exception of DEP and DETP in plasma (Table 1; Fig. S1). DETP, however, presented the best correlation between exposure and concentration in the matrix for both hair and urine (Table 1). As presented with the example of DEP (Fig. S1), inter-group differentiation based on metabolite concentration was best for urine, was acceptable for hair, and was quite poor for plasma, except for TCPy (Table 1; Figs. S2, S3, S4). Regarding the equivalent level of exposure, the highest concentration was observed for TCPy in the three matrices (Table 1).

### Pyrethroids

Although no parent pyrethroid was detected in urine, two out of the three pyrethroids to which animals were exposed were detected in hair and in plasma (Fig. 2). Cyhalothrin was detected from the 20 µg/kg exposure level in hair and from the 4 µg/kg exposure level in plasma. Cypermethrin was detected from the 200 µg/kg exposure level in hair and from the 10 µg/kg exposure level in plasma. Permethrin was only detected in plasma from 100 µg/kg. At equal level of exposure, the λ-cyhalothrin concentration in plasma was approximately 10 times higher than cypermethrin and approximately 100 times higher than permethrin, which could explain why permethrin was not detected in hair. The three pyrethroid metabolites tested in the current study were detected in all the exposure groups in the three matrices, except for 3-phenoxybenzoic acid (3-PBA) and 3-(2-chloro-3,3,3-trifluoro-1-propenyl)-2,2-dimethylcyclopropane carboxylic acid (ClCF_3_CA) in the urine of the control animals. Urinary metabolites presented a weaker association with the level of exposure than the hair and plasma concentrations (*R*_Pearson_) and comparable inter-groups differences. As for the metabolites of organophosphates, the urinary concentrations of 3-(2,2-dichlorovinyl)-2,2-dimethylcyclopropane-1-carboxylic acid (Cl_2_CA) and 3-PBA were not significantly different between the two highest levels of exposure. The highest concentration was observed for Cl_2_CA in hair and urine and for 3-PBA in plasma.

### Carbamates

The two carbamate metabolites, 2-IPP and carbofuran phenol, were detected in all of the animal groups in the three matrices, except for 2-IPP in the urine of the controls animals. Inter-group differentiation was better in the highest exposure groups in hair, whereas it was better in the lowest concentration levels in urine, and it was quite poor in plasma, whatever the level of exposure (Table 1; Figs. S2, S3, S4).

### Other pesticides

Fipronil and its metabolite fipronil sulfone were detected in all of the groups in hair and plasma, but the two compounds were not detected in the urine of the control animals, and fipronil was only detected from the 10 µg/kg exposure level. The two compounds presented good inter-group differentiation and a significant association between the concentration in the matrix and the level of exposure in the three matrices, although both *R*_Pearson_ and *R*_Spearman_ were higher for hair (Table 1). In the three matrices, the metabolite presented quite a higher concentration than the parent.

Trifluralin was detected in all of the groups, including controls, in hair, urine, and plasma, but the association between the concentration in the matrix and the level of exposure was quite better for hair and plasma than for urine. Similarly, the inter-group differentiation was quite easy when it was based on the hair and plasma concentrations, but was almost impossible with urine (Figs. S2, S3, S4). Diflufenican was detected in the hair of all of the animals, including the controls, from the 20 µg/kg exposure level in urine and from the first level of exposure in plasma. For diflufenican, the inter-group differentiation was best when it was based on the concentration in hair, followed by plasma, but it was rather poor when based on urine. Oxadiazon was detected in hair and plasma of all of the groups of exposure, with better inter-group differentiation in hair, but it was not detected in urine, whatever the level of exposure. Propiconazole was detected in the hair samples of all of the groups of exposure, but significant differences between adjacent groups were only observed between the three highest levels of exposure. Propiconazole was not detected in rat plasma and urine, whatever the level of exposure.

Adjusting the urinary concentration (ng/mL) with the volume of urine (mL) to obtain the amount excreted over 24 h (ng) decreased the correlation with the level of exposure for all the chemicals except for TCPy (*R* ^2^ = 0.7004 vs. 0.6802), DEP (*R* ^2^ = 0.7069 vs. 0.6712), DETP (*R* ^2^ = 0.7412 vs. 0.6845), Cl_2_CA (*R* ^2^ = 0.5956 vs. 0.5856), 3-PBA (*R* ^2^ = 0.6174 vs. 0.5676), and 2-IPP (*R* ^2^ = 0.6845 vs. 0.6750) for which the correlation was slightly increased. Similarly, adjusting the urinary concentration with the creatinine concentration had limited effects on both *R*_Pearson_ and *R*_Spearman_.

### Reverse classification analysis (RCA) and the number of detected pesticides

**Fig. 3:**
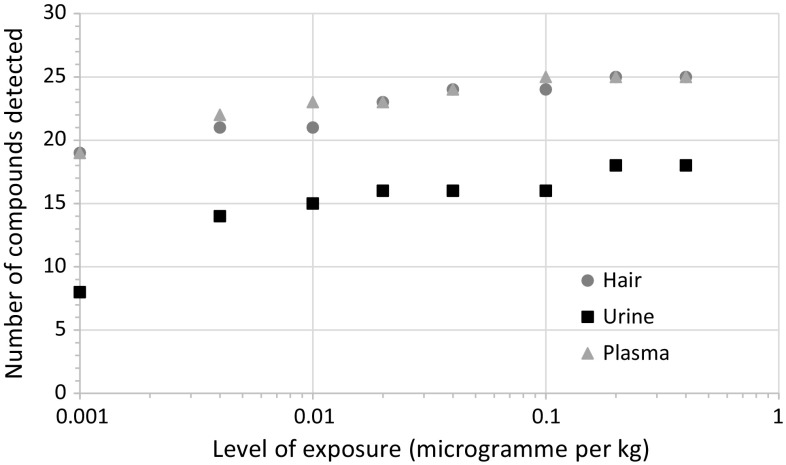
Number of chemicals detected in hair, urine, and plasma for the different levels of exposure

The RCA scores based on pesticide concentration in the matrix are presented in Fig. 2. Hair provided the best RCA scores for 13 out of the 27 target compounds: all of the organochlorines, fipronil, fipronil sulfone, diflufenican, oxadiazon, and propiconazole. Hair also provided the lowest number of compounds that were never detected, whatever the level of exposure (two out of 27). Urine allowed reaching the highest RCA scores for eight compounds but provided a real advantage over hair and plasma only for four of them: DEP, TCPy, 2-IPP, and carbofuran phenol. Moreover, urine presented the highest number of undetected compounds (nine out of 27). Plasma provided the highest RCA scores for three compounds (i.e., chlorpyrifos, cyhalothrin, and trifluralin), although close RCA values were obtained with hair for cyhalothrin and trifluralin. In plasma, among the 27 target compounds, only propiconazole and diazinon were not detected. The number of target chemicals detected in the biological matrices increased with increasing exposure, but was always higher in hair and plasma than in urine (Fig. 3). In control animals, 19 compounds were detected in hair and in plasma, and eight were detected in urine.

### Pigmentation

The influence of hair pigmentation on pesticide incorporation was assessed by comparing the concentration detected in white hair versus pigmented hair which were collected and analyzed separately from each animal. A slope close to the value of 1 indicated a poor influence of pigmentation on the compound concentration in hair (Table S2), as previously demonstrated in humans for other compounds (Appenzeller et al. 2007b). For the majority of the chemicals tested during the current study, pigmentation seemed to have a very limited effect on the concentration in hair, and only few compounds seemed to be pigmentation sensitive.

## Discussion

The present results definitely demonstrate that the concentration of pesticides and their metabolites in hair is representative of the level of exposure. For all of the chemicals detected in hair, the association between the exposure and the concentration in hair was always significant, with the poorest *p* value for *R*_Pearson_ equal to 0.000274 (observed for propiconazole). For most compounds, the association between the exposure intensity and the concentration of chemicals in hair was stronger than or comparable to urine and plasma. Although the association was better for nonpolar compounds such as organochlorines, it also concerned polar compounds such as metabolites of organophosphate and pyrethroid pesticides.

The ability to differentiate animals from different groups of exposure or to reattribute individuals to their correct group of exposure based on pesticide concentration in a matrix depended on both the matrix and the chemical. Hair analysis proved to be a highly efficient method for organochlorines and other parent pesticides, and also provided relevant results for other compounds, including polar metabolites of organophosphate pesticides and pyrethroids. As expected, urine was best adapted for detecting polar chemicals such as organophosphates and pyrethroid metabolites, and it also allowed acceptable results to be reached for some parent compounds, including some organochlorine pesticides, although urine is generally not considered for their analysis (Centers for Diseases Control and Prevention 2009). Urine, however, presented the highest rate of non-detected compounds compared to the two other matrices. Plasma allowed relevant information to be obtained regarding exposure to most compounds, with the exception of dialkyl phosphates for which this matrix proved to be unsuitable. Although blood is considered to be the compartment of choice for organochlorine biomonitoring (Centers for Diseases Control and Prevention 2009), organochlorine concentration in hair presented a better association with exposure and allowed better *R*_Pearson_ and *R*_Spearman_ values and RCA scores to be reached than with plasma.

The differences in pesticide concentrations observed between the urine and the plasma compartments are likely attributable to pesticide metabolism and physicochemical properties. For instance, the low concentration of dialkyl phosphate in plasma, whatever the level of exposure of animals, could be explained by the fast metabolization of the parent compound (0.2 h for chlorpyrifos) (Timchalk et al. 2007) followed by rapid transfer of these highly hydrophilic metabolites to urine. In contrast, the hydrophobic character of some compounds such as pyrethroids and to a lesser extent organochlorines (Chata et al. 2016) may limit their transfer from plasma to urine. In that regard, hair appears to be less affected by this phenomenon and contains both hydrophilic and hydrophobic compounds.

It has to be noticed that the matrices investigated during the current study actually have different temporal windows of detection. An analysis of biological fluids (i.e., urine and blood) is rather representative of recent exposure, especially for chemicals with short life spans such as pyrethroid metabolites (3-PBA: half-life of 4–5 h in rat blood after oral dosing of pyrethroid) (Starr et al. 2012) and organophosphate metabolites (DEP: half-life of 0.2 h for α-phase in rat blood after oral dosing of chlorpyrifos) (Timchalk et al. 2007). In contrast, hair is representative of periods covering weeks to months (Appenzeller and Tsatsakis 2012; Kintz et al. 2015). As a result, the protocol design of the current study, with an identical time lapse between gavage and fluid sampling for all of the animals, increased the apparent performances of urine and plasma analyses regarding representativeness of the exposure levels. In the case of random sampling time over the day, which is more representative of most epidemiological studies, the association between the pesticide concentration in the matrix and the level of exposure would have definitely been lower for plasma and urine because of intra-day variability, whereas it would not have affected results obtained from hair.

The presence of pesticides in controls was definitely not due to analytical contamination because the target compounds were always absent from the blank controls (solvent, extract without matrix) and was, therefore, really attributable to animal exposure (residual from the animal breeding center or the background level of exposure during experiments due to food, litter, or air), although these aspects were not further investigated during the current study. The presence of some chemicals in the control animals was not surprising because several compounds were reported to always be detected in humans (general population without specific exposure) (Salquebre et al. 2012) and also in control laboratory animals (Peiffer et al. 2013). The ability to document background exposure can be crucial, especially during experiments that are focused on low dose-related effects, because it may help to demonstrate (1) the exposure to the target compound(s) of controls that are supposedly not exposed and (2) the co-exposure to other chemicals that may induce combined effects. The two latter aspects may significantly bias interpretation of the results; therefore, these would need to be controlled. The current results of the study clearly highlight the relevance of hair, particularly over the choice of using urine for analysis for this purpose.

Although the influence of pigmentation on pesticide concentration in hair cannot be excluded, the current results demonstrated limited effects for most of the investigated pesticides. Because the range of concentrations of pollutants in hair within a population generally covers several orders of magnitudes (from the less exposed to the most exposed individuals), the differences observed during this study between samples of white and black hair are unlikely to induce significant misclassification of individuals according to their level of exposure. Moreover, the current situation (black hair with a high content of melanin vs. white hair, meaning absence of melanin) can be considered as an extreme scenario. The current study is therefore not representative of studies on humans, in which the percentage of individuals presenting totally non-pigmented hair is considered to be low, especially in children and pregnant women, which are favorite target populations for epidemiological studies focused on exposure and exposure-associated outcomes (Attflied et al. 2014; Burns et al. 2012; Castorina et al. 2010; Woodruff et al. 2011).

**Fig. 4:**
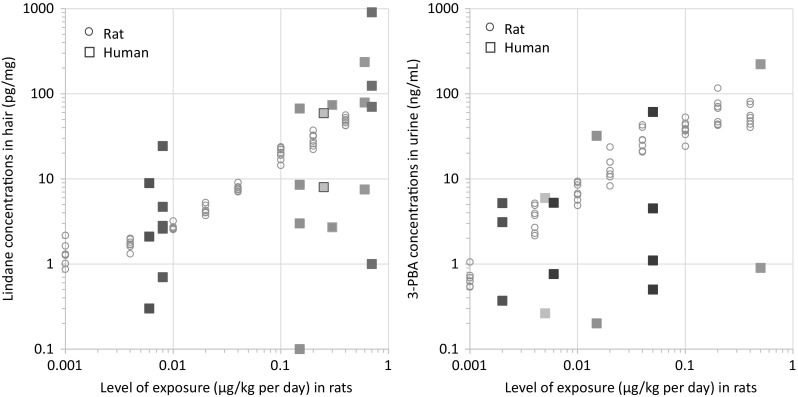
(*Left*) lindane concentrations in rat hair (the current work) and in human hair (reported from the literature). (*Right*) 3-PBA concentration in rat urine (the current work) and in human urine (reported from the literature). The data that correspond to the rats (*circles*) are related to the levels of exposure presented on the *x*-axis. The data that correspond to humans (*squares*) are not related to the level of exposure and are situated close to similar range of concentration observed in rats for better visibility. For humans, the *upper square* represents the highest concentration detected, and the *lower square* represents the lowest concentration detected (*Note*: When the lowest value was not detected, it was replaced by a half limit of detection). An *intermediate square* represents the median or the mean value (when available); *several intermediate squares* correspond to several sub-populations. Data on humans were obtained from many publications (Attflied et al. 2014; Behrooz et al. 2012; Castorina et al. 2010; Covaci et al. 2002, 2008; McKelvey et al. 2013; Oulhote and Bouchard 2013; Salquebre et al. 2012; Tsatsakis et al. 2008a, b; Wielgomas 2013; Wielgomas et al. 2012; Zhang et al. 2007) and from personal data

Although we conducted these experiments on an animal model, which is the only approach that allows for controlled exposure to pesticides, the purpose of the current study was definitely its transposition to human (with a view to classify individuals according to their level of exposure, or internal dose as final objective). We compared the pesticide concentration detected in biological matrices collected from the animals to data that were available in the literature for humans in the same matrices. Because information was not available for all of the pesticides tested in the current study, a comparison between humans and animals was focused on two chemicals for which large amounts of data are available in the literature. The two chemicals are lindane (organochlorine pesticide), which is to date among the most frequently analyzed chemicals in hair, and 3-PBA (pyrethroid metabolite), which is a frequently analyzed pesticide metabolites in urine. The range of concentrations of lindane in hair and 3-PBA in urine resulting from exposure of the animals was clearly comparable to concentration levels reported in the literature for humans (Fig. 4). We observed the lowest levels of lindane in human hair for inhabitants of Luxembourg (*n* = 14) (Salquebre et al. 2012) and Northern Poland (*n* = 40) (Wielgomas et al. 2012). The median (Luxembourg) and mean (Poland) concentration values were in the range of those detected in animals administered 4 µg/kg of lindane up to 20 µg/kg, three times a week. The highest concentration of lindane in hair was observed for a Greek rural population, including possible occupational exposure (Tsatsakis et al. 2008a, b), with median concentration values above those detected in animals submitted to the highest level of exposure of our experiments (400 µg/kg). The lowest concentration of 3-PBA in human urine (Fig. 4) involved a French pregnant women (unpublished data), with a median value close to what was detected in animals exposed to 4 µg/kg, three times a week. The highest concentration reported in the literature involved pregnant women living in the Salinas Valley in California (Castorina et al. 2010). The highest 3-PBA concentration in urine was above the concentration detected in the animals that were exposed to the highest level during our experiments.

In summary, the results of the current study demonstrate for the first time the significant association between the levels of exposure to pesticides and their resulting concentrations in hair. Even though we conducted this study on rats and used a limited number of pesticides, the hypothesis of similar behaviors for other chemicals and other species such as human seems to be reasonable. Regarding the transposition to humans, although pesticide concentration in hair does not yet allow for the determination of pesticide intake—at least because of interspecies differences—the current study still demonstrates that hair analysis allows reliable classification of individuals according to their respective level of exposure. This proof-of-concept, therefore, represents a step forward in considering hair analysis as a reliable tool to be used during epidemiological studies to investigate exposure-associated adverse health effects.

## Electronic supplementary material

Below is the link to the electronic supplementary material.

Supplementary material 1 (PPTX 121 kb)

Supplementary material 2 (PPTX 59 kb)

Supplementary material 3 (DOCX 12 kb)

Supplementary material 4 (DOCX 13 kb)
