# Supplementary material for: Hair analysis for the biomonitoring of pesticide exposure: comparison with blood and urine in a rat model
Source: Arch Toxicol. 2016 Dec 23;91(8):2813–25. doi: 10.1007/s00204-016-1910-9 (PMC5515982; doi:10.1007/s00204-016-1910-9)
Supplement: Supplementary file 1 — Supplementary material 1 (PPTX 121 kb) [file 204_2016_1910_MOESM1_ESM.pptx]

## Slide 1
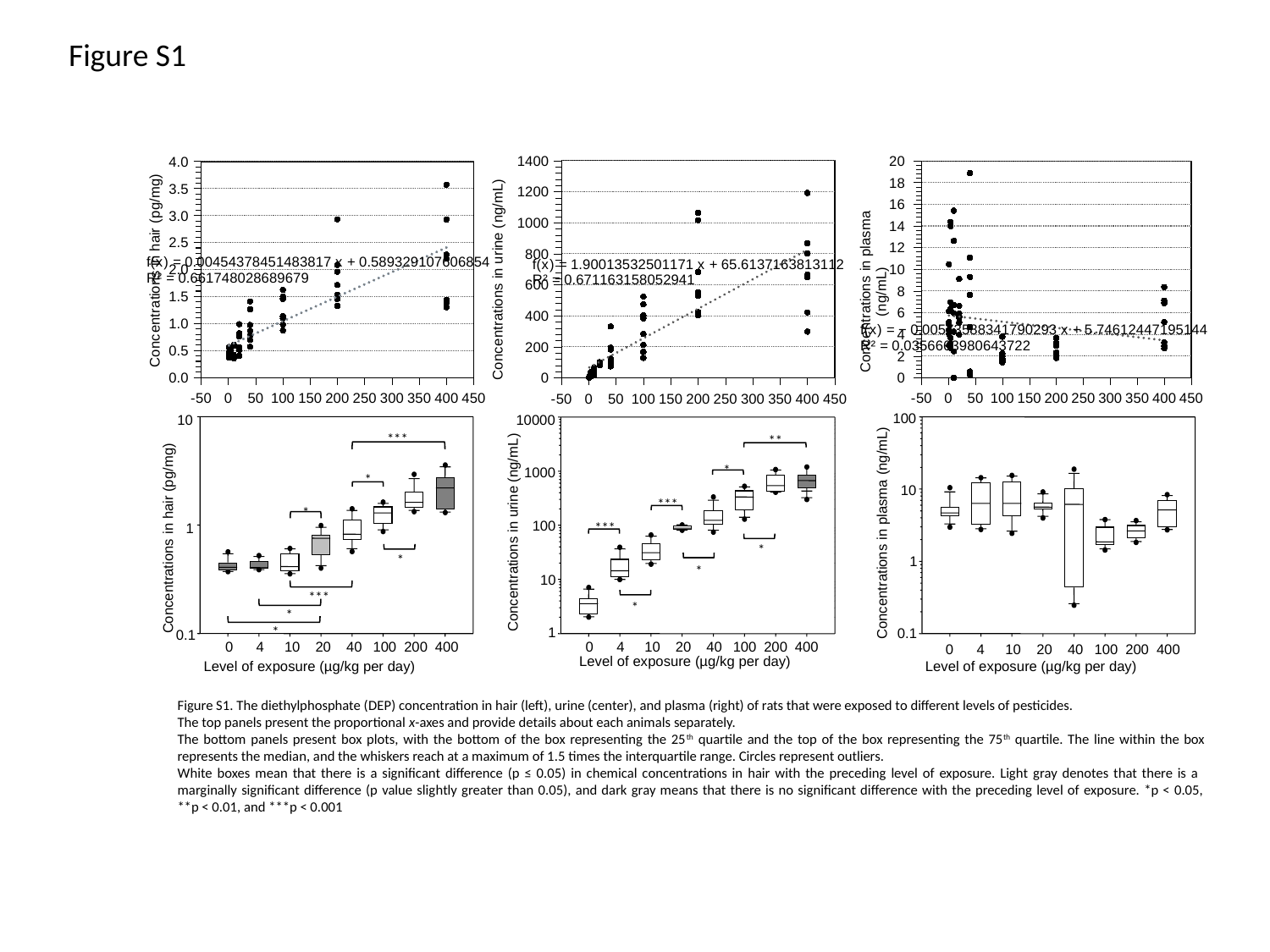

Figure S1
### Chart
| Category | plasma conc pg/mL |
|---|---|
### Chart
| Category | Urine adjusted (500 or 10) (ng/mL) |
|---|---|
### Chart
| Category | |
|---|---|100
10
Concentrations in plasma (ng/mL)
1
0.1
0
4
10
20
40
100
200
400
Level of exposure (µg/kg per day)
10
***
*
*
1
Concentrations in hair (pg/mg)
*
0.1
0
4
10
20
40
100
200
400
***
*
*
10000
**
*
***
***
*
*
*
0
4
10
20
40
100
200
400
1000
100
Concentrations in urine (ng/mL)
10
1
Level of exposure (µg/kg per day)
Level of exposure (µg/kg per day)
Figure S1. The diethylphosphate (DEP) concentration in hair (left), urine (center), and plasma (right) of rats that were exposed to different levels of pesticides.
The top panels present the proportional x-axes and provide details about each animals separately.
The bottom panels present box plots, with the bottom of the box representing the 25th quartile and the top of the box representing the 75th quartile. The line within the box represents the median, and the whiskers reach at a maximum of 1.5 times the interquartile range. Circles represent outliers.
White boxes mean that there is a significant difference (p ≤ 0.05) in chemical concentrations in hair with the preceding level of exposure. Light gray denotes that there is a marginally significant difference (p value slightly greater than 0.05), and dark gray means that there is no significant difference with the preceding level of exposure. *p < 0.05,
**p < 0.01, and ***p < 0.001
