# Supplementary material for: Hair analysis for the biomonitoring of pesticide exposure: comparison with blood and urine in a rat model
Source: Arch Toxicol. 2016 Dec 23;91(8):2813–25. doi: 10.1007/s00204-016-1910-9 (PMC5515982; doi:10.1007/s00204-016-1910-9)
Supplement: Supplementary file 2 — Supplementary material 2 (PPTX 59 kb) [file 204_2016_1910_MOESM2_ESM.pptx]

## Slide 1
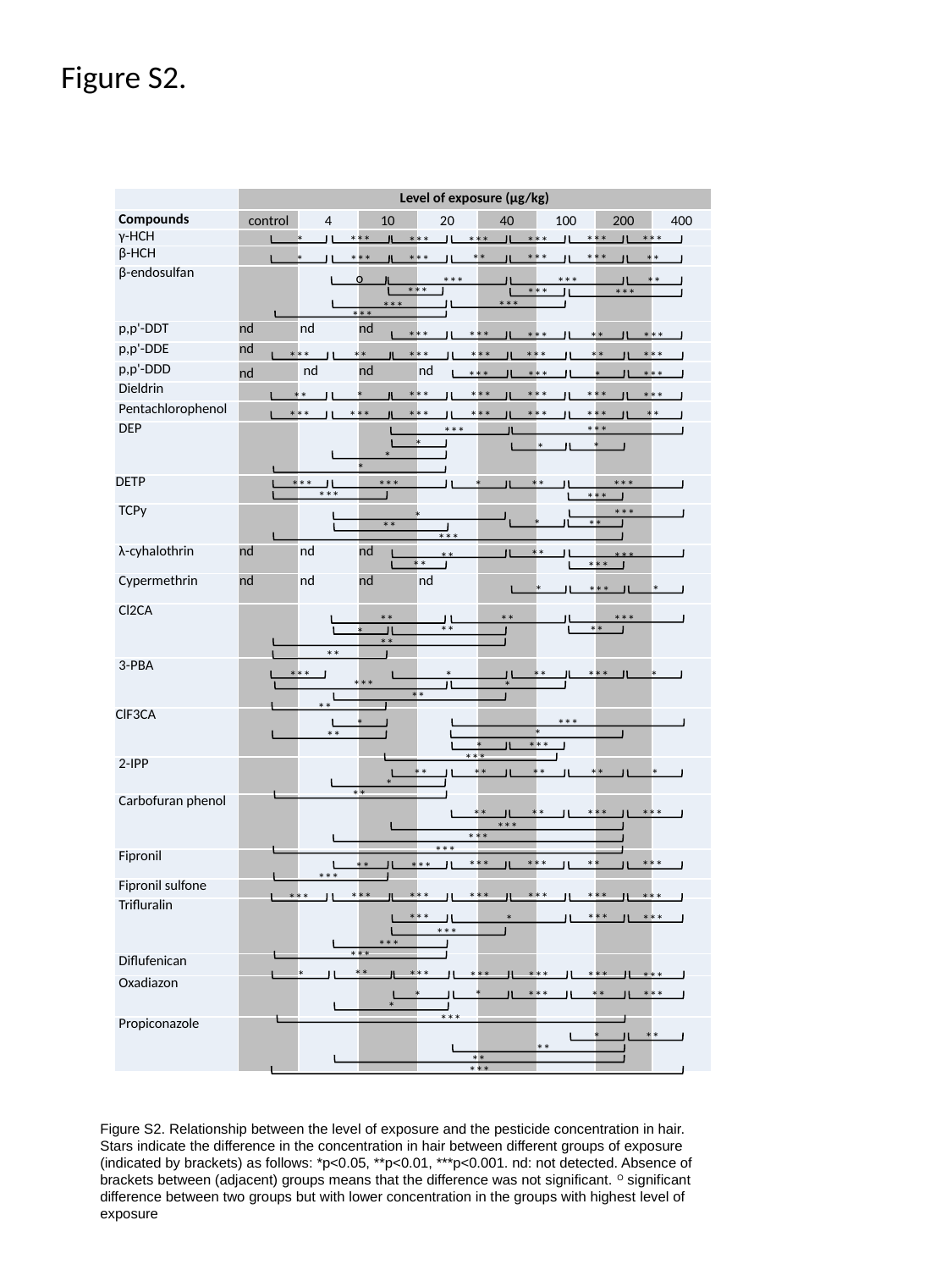

Figure S2.
| | Level of exposure (µg/kg) | | | | | | | |
| --- | --- | --- | --- | --- | --- | --- | --- | --- |
| Compounds | control | 4 | 10 | 20 | 40 | 100 | 200 | 400 |
| γ-HCH | | | | | | | | |
| β-HCH | | | | | | | | |
| β-endosulfan | | | | | | | | |
| p,p'-DDT | nd | nd | nd | | | | | |
| p,p'-DDE | nd | | | | | | | |
| p,p'-DDD | nd | nd | nd | nd | | | | |
| Dieldrin | | | | | | | | |
| Pentachlorophenol | | | | | | | | |
| DEP | | | | | | | | |
| DETP | | | | | | | | |
| TCPy | | | | | | | | |
| λ-cyhalothrin | nd | nd | nd | | | | | |
| Cypermethrin | nd | nd | nd | nd | | | | |
| Cl2CA | | | | | | | | |
| 3-PBA | | | | | | | | |
| ClF3CA | | | | | | | | |
| 2-IPP | | | | | | | | |
| Carbofuran phenol | | | | | | | | |
| Fipronil | | | | | | | | |
| Fipronil sulfone | | | | | | | | |
| Trifluralin | | | | | | | | |
| Diflufenican | | | | | | | | |
| Oxadiazon | | | | | | | | |
| Propiconazole | | | | | | | | |
*
***
***
***
***
***
***
**
***
***
***
***
**
*
o
**
***
***
***
***
***
***
***
***
***
***
***
**
***
***
***
**
**
***
***
***
***
***
***
*
***
***
***
*
***
***
**
***
***
***
***
***
**
***
***
***
*
*
*
*
*
***
***
*
**
***
***
***
***
*
*
**
**
***
**
**
***
**
***
*
*
***
**
***
**
**
**
*
**
**
**
*
***
***
*
***
*
**
**
***
*
*
**
*
***
***
**
**
**
*
**
*
**
**
***
***
**
***
***
***
***
**
***
***
***
**
***
***
***
***
***
***
***
***
***
***
***
*
***
***
***
**
*
***
***
***
***
***
*
*
***
**
***
*
***
*
**
**
**
***
Figure S2. Relationship between the level of exposure and the pesticide concentration in hair. Stars indicate the difference in the concentration in hair between different groups of exposure (indicated by brackets) as follows: *p<0.05, **p<0.01, ***p<0.001. nd: not detected. Absence of brackets between (adjacent) groups means that the difference was not significant. O significant difference between two groups but with lower concentration in the groups with highest level of exposure

## Slide 2
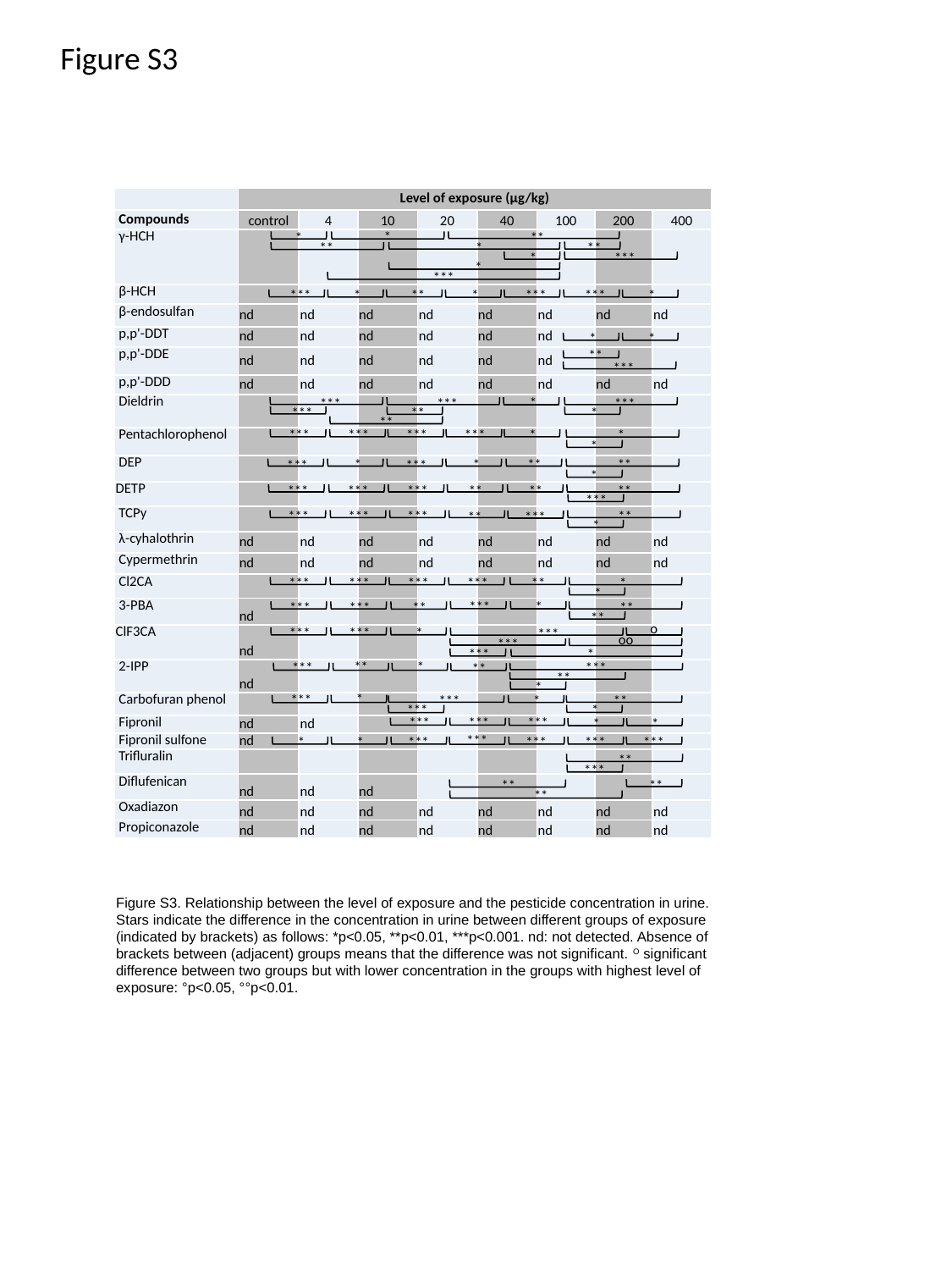

Figure S3
| | Level of exposure (µg/kg) | | | | | | | |
| --- | --- | --- | --- | --- | --- | --- | --- | --- |
| Compounds | control | 4 | 10 | 20 | 40 | 100 | 200 | 400 |
| γ-HCH | | | | | | | | |
| β-HCH | | | | | | | | |
| β-endosulfan | nd | nd | nd | nd | nd | nd | nd | nd |
| p,p'-DDT | nd | nd | nd | nd | nd | nd | | |
| p,p'-DDE | nd | nd | nd | nd | nd | nd | | |
| p,p'-DDD | nd | nd | nd | nd | nd | nd | nd | nd |
| Dieldrin | | | | | | | | |
| Pentachlorophenol | | | | | | | | |
| DEP | | | | | | | | |
| DETP | | | | | | | | |
| TCPy | | | | | | | | |
| λ-cyhalothrin | nd | nd | nd | nd | nd | nd | nd | nd |
| Cypermethrin | nd | nd | nd | nd | nd | nd | nd | nd |
| Cl2CA | | | | | | | | |
| 3-PBA | nd | | | | | | | |
| ClF3CA | nd | | | | | | | |
| 2-IPP | nd | | | | | | | |
| Carbofuran phenol | | | | | | | | |
| Fipronil | nd | nd | | | | | | |
| Fipronil sulfone | nd | | | | | | | |
| Trifluralin | | | | | | | | |
| Diflufenican | nd | nd | nd | | | | | |
| Oxadiazon | nd | nd | nd | nd | nd | nd | nd | nd |
| Propiconazole | nd | nd | nd | nd | nd | nd | nd | nd |
*
**
*
*
**
**
***
*
*
***
*
**
***
*
***
*
***
*
*
**
***
*
***
***
***
***
*
**
**
*
***
***
***
***
*
*
*
*
**
**
***
***
*
**
**
**
***
***
***
***
***
***
***
**
***
**
*
***
**
***
*
***
***
*
*
***
***
**
***
**
**
o
*
***
***
***
oo
***
*
***
**
*
***
***
**
**
*
*
***
*
***
**
***
*
***
***
***
*
*
***
*
*
***
***
***
***
**
***
**
**
**
Figure S3. Relationship between the level of exposure and the pesticide concentration in urine. Stars indicate the difference in the concentration in urine between different groups of exposure (indicated by brackets) as follows: *p<0.05, **p<0.01, ***p<0.001. nd: not detected. Absence of brackets between (adjacent) groups means that the difference was not significant. O significant difference between two groups but with lower concentration in the groups with highest level of exposure: °p<0.05, °°p<0.01.

## Slide 3
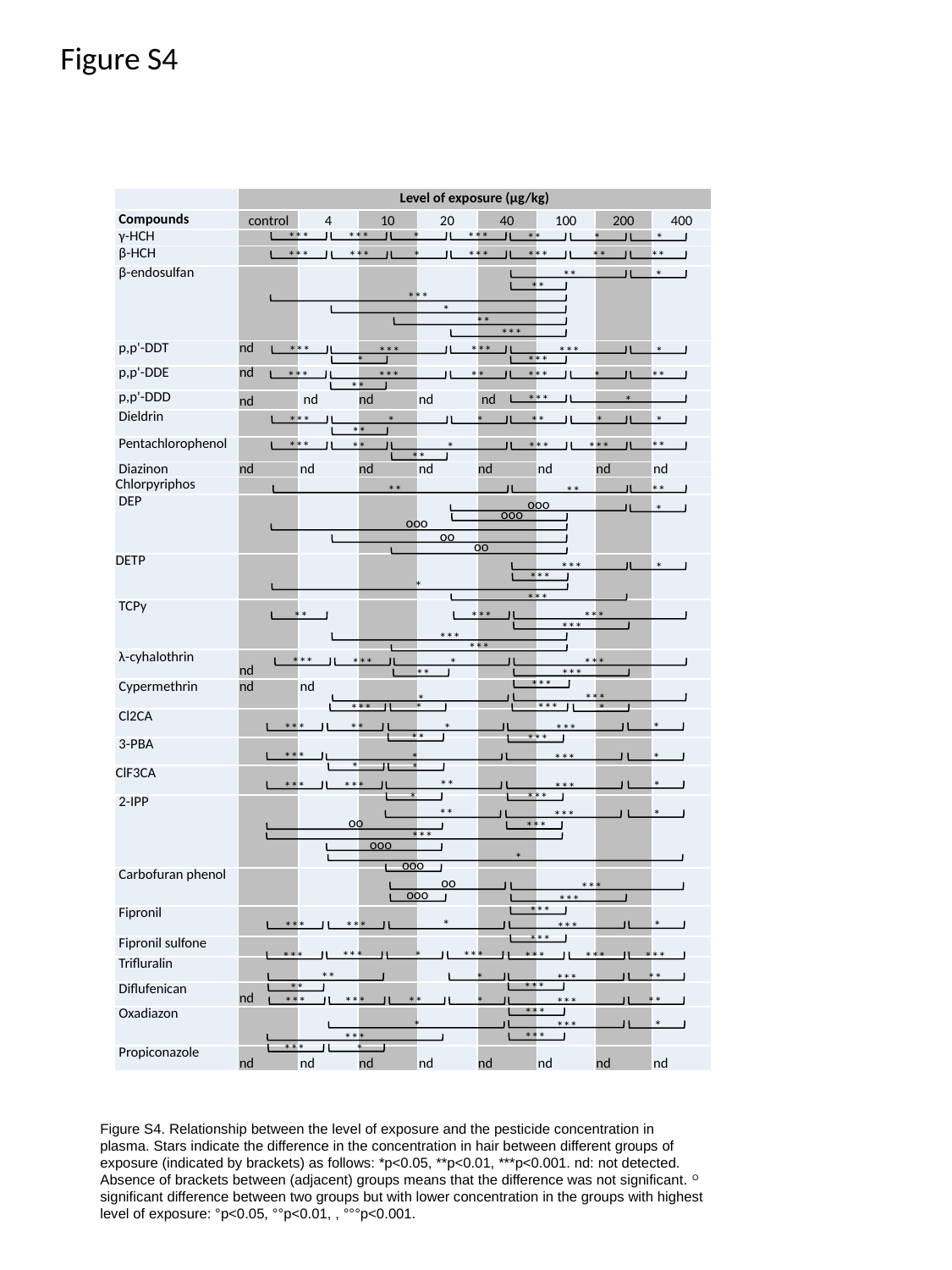

Figure S4
| | Level of exposure (µg/kg) | | | | | | | |
| --- | --- | --- | --- | --- | --- | --- | --- | --- |
| Compounds | control | 4 | 10 | 20 | 40 | 100 | 200 | 400 |
| γ-HCH | | | | | | | | |
| β-HCH | | | | | | | | |
| β-endosulfan | | | | | | | | |
| p,p'-DDT | nd | | | | | | | |
| p,p'-DDE | nd | | | | | | | |
| p,p'-DDD | nd | nd | nd | nd | nd | | | |
| Dieldrin | | | | | | | | |
| Pentachlorophenol | | | | | | | | |
| Diazinon | nd | nd | nd | nd | nd | nd | nd | nd |
| Chlorpyriphos | | | | | | | | |
| DEP | | | | | | | | |
| DETP | | | | | | | | |
| TCPy | | | | | | | | |
| λ-cyhalothrin | nd | | | | | | | |
| Cypermethrin | nd | nd | | | | | | |
| Cl2CA | | | | | | | | |
| 3-PBA | | | | | | | | |
| ClF3CA | | | | | | | | |
| 2-IPP | | | | | | | | |
| Carbofuran phenol | | | | | | | | |
| Fipronil | | | | | | | | |
| Fipronil sulfone | | | | | | | | |
| Trifluralin | | | | | | | | |
| Diflufenican | nd | | | | | | | |
| Oxadiazon | | | | | | | | |
| Propiconazole | nd | nd | nd | nd | nd | nd | nd | nd |
***
*
***
***
**
*
*
***
***
*
***
**
**
***
**
*
**
***
*
**
***
***
***
***
*
***
***
*
***
***
**
*
**
***
**
***
*
***
*
**
*
*
*
**
**
***
**
***
***
*
**
**
**
**
ooo
*
ooo
ooo
oo
oo
***
*
***
*
***
**
***
***
***
***
***
***
***
*
***
**
***
***
***
*
*
***
***
*
*
***
**
*
***
**
***
***
*
*
***
*
*
**
*
***
***
***
*
***
**
*
***
oo
***
***
ooo
*
ooo
oo
***
ooo
***
***
*
*
***
***
***
***
***
***
*
***
***
***
***
**
*
**
***
***
**
***
**
*
**
***
***
***
*
*
***
***
***
*
***
Figure S4. Relationship between the level of exposure and the pesticide concentration in plasma. Stars indicate the difference in the concentration in hair between different groups of exposure (indicated by brackets) as follows: *p<0.05, **p<0.01, ***p<0.001. nd: not detected. Absence of brackets between (adjacent) groups means that the difference was not significant. O significant difference between two groups but with lower concentration in the groups with highest level of exposure: °p<0.05, °°p<0.01, , °°°p<0.001.
