# Supplementary material for: Hair analysis for the biomonitoring of pesticide exposure: comparison with blood and urine in a rat model
Source: Arch Toxicol. 2016 Dec 23;91(8):2813–25. doi: 10.1007/s00204-016-1910-9 (PMC5515982; doi:10.1007/s00204-016-1910-9)
Supplement: Supplementary file 3 — Supplementary material 3 (DOCX 12 kb) [file 204_2016_1910_MOESM3_ESM.docx]

| **Table S1. Pesticides administrated to the rats with metabolites and an indication of the *target chemicals (parent and metabolites) analyzed in hair, urine and plasma** | | |
| --- | --- | --- |
| **Chemical class** | **Exposure pesticides** | **Metabolites analyzed** |
| Organochlorines | *γ-HCH (lindane) |  |
|  | *β-HCH |  |
|  | * β-endosulfan |  |
|  | *p,p’-DDT | *p,p’-DDE, *p,p’-DDD |
|  | *p,p’-DDE | *p,p’-DDD |
|  | *Dieldrin |  |
|  | *Pentachlorophenol |  |
| Organophosphates | *Diazinon | *DETP, *DEP |
|  | *Chlorpyrifos | *TCPy, *DETP, *DEP |
| Pyrethroids | *Cyhalothrin | *ClCF3CA, *3-PBA |
|  | *Permethrin | *Cl_2_CA, *3-PBA |
|  | *Cypermethrin | *Cl_2_CA, *3-PBA |
| Azole | *Propiconazole |  |
| Phenylpyrazole | *Fipronil | *Fipronil sulfone |
| Oxadiazin | *Oxadiazon |  |
| Carboxamide | *Diflufenican |  |
| Anilin | *Trifluralin |  |
| Carbamates | Carbofuran | *Carbofuran phenol |
|  | Propoxur | *2-IPP |
| *Target chemicals |  |  |
