# Supplementary material for: Hair analysis for the biomonitoring of pesticide exposure: comparison with blood and urine in a rat model
Source: Arch Toxicol. 2016 Dec 23;91(8):2813–25. doi: 10.1007/s00204-016-1910-9 (PMC5515982; doi:10.1007/s00204-016-1910-9)
Supplement: Supplementary file 4 — Supplementary material 4 (DOCX 13 kb) [file 204_2016_1910_MOESM4_ESM.docx]

| **Table S2. Association between the concentration in white hair and concentration in pigmented hair** | | |
| --- | --- | --- |
| Compounds | Slope | R^2^ |
| **Organochlorines** |  |  |
| γ-HCH | 0.969 | 0.984 |
| β-HCH | 1.021 | 0.973 |
| β-endosulfan | 0.965 | 0.761 |
| p,p'-DDT | 0.876 | 0.893 |
| p,p'-DDE | 1.287 | 0.956 |
| p,p'-DDD | 0.673 | 0.716 |
| Dieldrin | 1.000 | 0.952 |
| Pentachlorophenol | 0.705 | 0.929 |
| **Organophosphates** | |  |
| Diazinon | NA | NA |
| Chlorpyriphos | NA | NA |
| DEP | 0.892 | 0.743 |
| DETP | 0.608 | 0.774 |
| TCPy | 0.435 | 0.694 |
| **Pyrethroids** |  |  |
| Permethrin | NA | NA |
| λ-cyhalothrin | 1.079 | 0.622 |
| Cypermethrin | 0.495 | 0.6714 |
| Cl_2_CA | 0.498 | 0.945 |
| 3-PBA | 1.070 | 0.776 |
| ClCF_3_CA | 0.220 | 0.185 |
| **Carbamates** |  |  |
| 2-IPP | 0.692 | 0.690 |
| Carbofuran phenol | 1.109 | 0.795 |
| **Others** |  |  |
| Fipronil | 1.017 | 0.947 |
| Fipronil sulfone | 1.114 | 0.964 |
| Trifluralin | 0.688 | 0.584 |
| Diflufenican | 0.937 | 0.958 |
| Oxadiazon | 0.860 | 0.800 |
| Propiconazole | 0.825 | 0.454 |
| Note: NA = not applicable; the compound was not detected in hair. | | |
